# On the Pathology and Treatment of Hydrocephalus

**Published:** 1824-09

**Authors:** R. Venables


### ART. II.—*On the Pathology and Treatment of Hydrocephalus.* By R. VENABLES, Esq.

NOTWITHSTANDING the numerous researches and the labours of various anatomists and physiologists, we still find the pathology of hydrocephalus involved in impenetrable obscurity. Some maintain that it is a disease of debility, and that the appropriate treatment consists in a judicious selection, and a well-regulated course, of tonics; while there are others, who as strenuously insist upon the inflammatory nature of this disease, and the necessity of active antiphlogistic measures for its relief.

Dr. Cheyne, in his first Essay on Hydrocephalus, (page 34,) observes, "To conclude, hydrocephalus appears to consist in a diseased action of a peculiar kind; but of what kind, we can as little explain as we can the nature of the scrofulous or syphilitic action. One object, therefore, here, as in these diseases, is to register and arrange every essential fact; and never to relax in our inquiry, until, by this induction, we shall arrive at a successful method of practice."

Dr. Cheyne states, 1st, that the disease often arises after a manifest disorder of the digestive organs has existed for a considerable time. 2dly. That the symptoms of the disease, when of short standing, often disappear while we are correcting the state of these organs. Hence he says, "Are we not justified by every analogy in stating, as a thing probable in the highest degree, that hydrocephalus often arises from the brain sympathising with the disordered condition of the liver and alimentary canal."*

It is one of the misfortunes of our science, that the operations of the component parts of our structure are so involved in mystery and obscurity, that all our inferences, as well as the principles of our practice, are equally doubtful. Dr. Cheyne applies the principle of sympathy to explain an unintelligible fact. We observe that the disease of one organ frequently, during its progress, involves other parts; and, in such cases, the secondary disease is said to arise from sympathy. But what this sympathy is, or wherein it consists, none have as yet satisfactorily explained.

Dr. Cheyne asserts, 3dly, as an established fact, that, "in its first stage, hydrocephalus is evidently attended with a considerably increased arterial action."† Is it not a much more intelligible mode of explaining the pathology of hydrocephalus, when consecutive to alimentary derangement, to refer the morbid state of the brain to the generally increased momentum of the circulation, affecting more particularly the weaker structures? Is sympathy a certain relation between similar or dissimilar structures? If it be, why do we find its diseases as variable, not only in their nature, but in the parts which they affect, as it is possible to imagine.

We can readily conceive how improper diet may, by producing a vitiated chyle, so far alter the quality of the blood, as to render it unfit for the purposes of health. Thus, the excitability of various organs may be preternaturally increased, or diminished. We also know that the different parts of our system are not all equally susceptible of excitement, from the application of the same stimuli. Let us apply this reasoning to explain the pathology of hydrocephalus. A secondary effect of improper diet, is a morbid excitability of the heart: hence the momentum of the blood is preternaturally increased, and an increased afflux of blood to the different parts is a consequential effect. If the strength and vigour of these parts be such as to resist preternatural distention, or to dispose of the increased quantity of blood thus propelled to them, their healthy structure remains unimpaired. But if any, either from original conformation, or from the ravages of disease, should prove too weak for this activity of excitement, such part will suffer and manifest symptoms of disease; while the others are but little, if at all, affected.

I am well convinced that many of the diseases which are now considered sympathetic, might be thus satisfactorily, but, at all

* *Essay I.* p. 35. † *Ibid.*

events, far more philosophically, explained.* If secondary diseases arose from sympathy, we should always find the same primary followed by the same secondary: as, for instance, hepatitis, when producing a secondary or consecutive disease, would be always followed by hydrocephalus, or pulmonary consumption, or by some one invariable and constant morbid affection. Experience, however, fully confutes this doctrine; and we find that hepatitis is followed, in some instances by hydrocephalus, in others by pulmonary irritation, or by ascites, anasarca, or cutaneous affections, according to the condition of these structures.

I have, in a work† which I have recently published, endeavoured to establish these principles; and, in illustration and further confirmation of them, I shall now submit the following case, interesting from its history, and the morbid appearances on dissection.

William Soundy, aged twelve years, a thin, emaciated boy, of a scrofulous habit. His sister died of what was supposed to be consumption.

July 16th, 1824.—I was requested to visit the above-named lad, and found him in the very last stage of emaciation; he was almost a perfect skeleton. When I saw him first, he was lying on a sofa, extremely irritable and peevish. He complained of severe pain in his head, and had complained much of this symptom for some considerable time. The eyes were clear, rather brilliant, the pupils natural in size, and perfectly sensible to light. There was a good deal of febrile heat, with a quick, frequent pulse, which, though hard, was still very small and thready. The respiration, when quiet, was nearly natural; but, upon agitation or exertion, became somewhat hurried. The bowels were sometimes relaxed, but, generally speaking, they were costive. The stools were occasionally approaching to a dark colour; they commonly consisted of a frothy mucus, mixed with a gelatinous slime. The urine was small in quantity, and high coloured; sometimes depositing a pink-coloured sediment. The skin harsh and dry; seldom any degree of moisture upon it. The tongue naturally moist, but of a cherry-red colour, with here and there a flake of a kind of sodden whitish appearance.

He did not complain of cough, and, while at rest, he experienced nothing of this sort; but exertion or agitation now and then was followed by a dry, husky cough. I inquired, but could not learn, that he had ever spit any blood, or expectorated much.

---

* Vide *Clinical Report on Dropsies*, by the Author.

† *Ibid.*

This boy had been ill for more than twelve months, and I was informed that the medical gentleman, who had attended him in the commencement of his illness, supposed him to be in a consumption.* He was under this gentleman's care till February last, when he spontaneously ceased to visit him longer. Of the previous treatment I know nothing; but his mother told me that, from the period in which the medical attendant gave up visiting him, she herself occasionally gave him magnesia and such mild medicines.

As the alvine evacuations were somewhat discoloured, I determined to try small and unirritating doses of mercury; and, as he appeared to labour under considerable irritation of both mind and body, I thought it prudent to combine the mercurial with narcotics. The following medicine was prescribed:— ℞. Pil. Hyd. ℈j. Ipecacuanha gr. xij. Ext. Hyosciami, Ext. Lactucæ utriusque gr. x. M. fiat pil. xij. Sumat ij. bis inter hepdomad.— ℞. Pulv. Rhei ℈j. Sulph. Potassæ ʒss. M. Divid. in pulv. xij. equal. Sumat j. bis in die pauxillo sacchari.

17th and 18th.—Little or no alteration in the symptoms; but on this day (18th) he took to bed, and did not get up.—Perstat.

19th.—As before; bowels have not been opened.—Injiciatur enema commune q. p.

20th.—The enema brought away a scanty, frothy, mucous motion. The symptoms no way abated; no thirst; appetite bad; seems unwilling to make the slightest exertion.—Perstat.

21st.—Thinks himself somewhat better.—Cont. u. a.

22d.—Not quite so well; bowels costive.—Repet. enema.

23d.—Bowels were gently moved, but the evacuations were no way healthy. The pain in the head had been very severe yesterday. Delirium and muttering, and some degree of coma, has been observed.

From the scrofulous appearance of this boy, and the evident signs of it in the other branches of his family, I determined (certainly without the slightest expectation of success,) to give the periodide of mercury; a preparation which I am inclined to report favourably of, as a remedy against scrofula. As there was a manifest tendency to coma, and as he was much averse to pills and powders, I gave him the periodide in solution.— ℞. Hyd. Period. gr. x. Solutionis Hydriod. Potassæ ℥ss. Aquæ distillata ℥vj. M. fiat solutio cujus sumat coch. j. mederter quaterve in die.†

* As I did not see the gentleman, I cannot answer for this point: however, I have since learned, from other sources, that this was his impression.

† I believe no other practitioner in England has used these preparations; nor was I aware that they had been tried by any other in Europe, till I saw it announced, in the Edinburgh Medical and Surgical Journal, that Professor Biera has been giving them in scrofulous diseases. It is satisfactory to me to find that

From this period he became completely insensible; the breathing being sometimes quite stertorous, at other times more easy and tranquil. The eyes became fixed, with a dull, filmy appearance. The pupils neither contracted nor dilated, but were wholly insensible to the stimulus of light. He died on the 26th, at four P.M.

### *Dissection, on the 28th.*

On opening the cranium, the skullcap was found to adhere more firmly than natural to the dura mater. The vessels on the exterior surface of this membrane were highly turgid with blood, and traces of increased vascular action were discoverable on the surface. On laying open the superior longitudinal sinus, two or three flakes of coagulable lymph were found loosely floating in the cavity. On raising the dura mater, the internal surface of this membrane was adherent in several places to the pia mater, at the point where the superior parts of the cerebrum touch the triangular space formed by the falx dipping down perpendicularly between the two hemispheres, and the dura mater extending itself over the brain. These connexions, which were formed of a strong ligamentous-like substance, were

---

the Professor attributes to these preparations the same chemical properties which I had discovered them to possess. The periodide of mercury, which may be obtained by decomposing a solution of corrosive muriate of mercury by one of hydriodate of potass, in the proportions of eight to ten, is a brilliant red powder, insoluble in water, but soluble in alcohol, ether, rectified spirits of turpentine, (I believe, essential oils,) and alkaline hydriodates. When prosecuting my experiments upon these substances, I discovered that, by triturating calomel and hydriodate of potass, a double decomposition takes place; and thus I have been in the habit of forming periodide of mercury by prescription.

The hydriodate of potass is an excellent test of the presence of corrosive sublimate. I shall take the liberty of subscribing from my manuscript notes, the observations which I made upon this subject:

"It occurred to me that the hydriodate of potass might be easily and readily applied to detect corrosive muriate of mercury intermixed with calomel. With this view, I obtained a specimen of calomel, and having weighed out a drachm, I put it into a Florence flask; and having poured over it half a pint of distilled water, I heated it to the boiling point. I then took it from over the lamp, and threw the whole upon the filter. To the filtered liquor I added a drop or two of diluted solution of hydriodate of potass; but no precipitation took place.

"Having dried the calomel, I weighed out a grain of corrosive muriate of mercury, and having divided it as equally as I could, I threw away one half, and mixed the remaining portion with the drachm of calomel. This mixture I put into a Florence flask, and having poured on half a pint of distilled water, I elevated the temperature, as before, to nearly the boiling point. The mixture was now poured on the filter. Upon adding a drop or two of a very weak solution of hydriodate of potass to two ounces of the filtered liquor, distinct traces of the periodide of mercury were discoverable, by the precipitation of a red powder. The necessity of a very diluted solution with such a minute quantity must be evident, when it is recollected that the periodide of mercury is soluble in an excess of the precipitating fluid.

"The hydriodates of iron and zinc, which are readily formed by decomposing the sulphates of these metals by an alkaline of hydriodate, I have found excellent tonics in scrofula."

very firm, and, on using sufficient force to break them, tore up the pia mater.

The vessels of the pia mater, both arterial and venous, were enlarged, and turgid with a dark-coloured blood. The vascular activity which had prevailed here was very evident.

The substance of the cerebrum itself was very soft and pulpy, and showed considerable vascularity through its whole substance. The fornix quite soft; and the foramen commune anticus so enlarged, as nearly to admit my thumb to pass through. The ventricles were very much enlarged, and distended with fluid. On the first opening of the dura mater, about an ounce and a half of fluid escaped; and, during the dissection, five ounces, three drachms, and nearly fifteen minims,* were collected. The plexus choroides was very vascular, and flakes of coagulable lymph adherent to it. Coagulable lymph was exuded on the base of the brain, at the junction of the optic nerves, and extending to the pons Varolii, and over the medulla oblongata. The cerebellum was very vascular, and firm in its structure. The ventricles seemed all to communicate, their cavities being morbidly enlarged.

*Appearances in the Abdomen.*—On opening the cavity of the abdomen, the peritoneum, omentum, and viscera, seemed healthy. The liver was natural in size and appearance. At the back part of the right hypochondrium, however, it was found adherent to the peritoneal coat of this cavity by an adventitious membrane from inflammation. The pancreas was hardened and tuberculated: in some parts the substance, when cut into, was cheesy. The spleen was natural in size and appearance; but there were extensive adhesions between it and the peritoneum lining the left hypochondre. The small intestines contained soft fæces; but in the colon there were small lumps of hardened or indurated fæces. The mucous membrane of the small intestines more vascular in appearance than natural; externally, they seemed quite healthy. The mesenteric glands were in some places hard, in others soft; and one seemed to contain a softish matter, of the consistence of very soft butter. The kidneys healthy, as also the urinary bladder.

*Appearances in the Chest.*—Pleura pulmonalis adhering firmly

---

* It may seem as if the quantity is stated with a degree of minuteness not to be attained. In the eighth Dissection, page 156, of Dr. Cheyne's "Essay on Hydrocephalus," he observes, "Rather more fluid than natural in the ventricles; but, with respect to this point, there was some difference of opinion." There is nothing more easy than to determine, with the utmost precision and accuracy, the exact quantity. A common glass tube, blown into a bulb or hollow ball in the centre, by applying the mouth, and thus exhausting the air, will take up the last drop, which may then be measured in graduated glasses. This was the plan which I adopted in the present instance, and which I have adopted for some time.

and intimately to the pleura costalis; lungs discoloured, hard, dense, like hepatized lungs, and, for the greater part, impermeable either to air or blood,—to the latter especially. The heart small; the substance of the ventricles very pale; the right containing blood, and frothy blood in the left ventricle. Nothing remarkable with respect to the pericardium.

The spine was in no degree incurvated; nor were the joints enlarged.

There can be little doubt that death ensued from the disorganization discovered to have taken place in the brain. But an important question here presents for our consideration: Was the disease of the brain a primary, or merely a consecutive, affection? On opening the thorax, the lungs were found very much diseased, and to such a degree as would have been sufficient to account for the fatal event, had not the state of the brain been such as to render it attributable rather to the disorganized condition of this organ. Yet I am inclined to think that the hydrocephalus, and general morbid condition of the brain, was a secondary affection, consequent to the diseased state of the lungs. I presume the primary source of ill health to have arisen in the digestive organs. Obstruction of the mesenteric glands, with an irritable and inflammatory condition of the mucous membrane of the small intestines, by inducing fever and increased vascular action, caused a greater afflux of blood to the pulmonary organs, than what their delicate structure* could support. This increased afflux of blood, in time, brought on languid inflammatory action, terminating ultimately in thickening of the pulmonary tissue, and gradual hepatization of these organs. Hence the circulation through the lungs became difficult, and the return of the blood from the brain was thus impeded. The pressure arising from the obstructed blood led to absorption and softening of the brain, and the vessels being turgid, took on the inflammatory action, as evidenced by the adhesions and layers of coagulable lymph found in different parts of the brain.

The fluid, too, which was taken from the ventricles was coagulable in toto: the whole becoming perfectly solid on being heated, without leaving the least watery residue, seems to indicate the inflammatory nature of its source.

Though the signs of inflammation in the abdomen were comparatively trifling, and of little amount, yet they were fully sufficient to justify the pathological principles just now advanced: add to which, that they were evidently traces of an early inflammatory process, which had ceased for some time.

* From the scrofulous tendency, I conceive it a fair inference that the pulmonary, as well as the cerebral structure, was weaker than what is consistent with health.

As far as I could learn, the complaint began in the bowels. At first there was little or no pain in the head, and the medical attendant looked upon the disease as pulmonary consumption:* therefore, we must take it for granted, that there could have been no symptoms whatever indicative of disease in the brain.

From the duration of the illness, (upwards of twelve months,) we may fairly assume that the brain was not originally the seat of disease; as, had it been a case of primary hydrocephalus, the symptoms would have been not only less equivocal, but no doubt infinitely more violent. Even when it came under my care, there was no vomiting, no nausea, nor any affection of the eye, which could have led to the presumption of water in the head; and yet, on dissection, nearly half a pint of fluid was found in the ventricles. Nor could it have been a sudden effusion of fluid;† because the soft and pulpy state of the brain, and the preternaturally enlarged state of the ventricles, fully showed that the accumulation of fluid was a slow and gradual process. The inflammatory adhesions, too, were evidently of some standing, and by no means of recent occurrence. The venous plethora, the arterial turgescence, and increased vascularity, are all objects of serious reflection for the pathologist. Upon the whole, I cannot but think that the disorder commenced in the alimentary organs; then the lungs became involved; and, when they became impermeable, so as that the blood could not circulate freely, turgescence of the vessels of the brain succeeded, inducing an accumulation of fluid, which at last proved fatal. The dark appearance of the vascular tissue of the brain, still further favours the foregoing view of the pathology of this case.

The influence which a morbid state of the digestive organs may exert in exciting hydrocephalus, is by no means a new idea. Drs. Cheyne and Yeats have shewn a great deal of zeal in this inquiry, and their labours have not been in vain. Dr. Cook, in the first volume of his "Treatise on Nervous Diseases," page 384, observing on the opinion of Dr. Yeats, that hepatic torpor is frequently a source of hydrocephalus, says, in a note, "It may be doubted, however, whether the affection of the brain be the consequence or the cause of the derangement of the functions of the stomach, liver, and intestines."

Be this as it may, they should never be neglected, because their re-action on the system in general, and on the weaker organ in particular, should be counteracted. In the present

* I do not know the symptoms upon which such a diagnosis was formed. I was given to understand, that there was neither cough, nor expectoration, nor any hæmoptoë.

† It will be recollected that, on the 23d, the patient was comatose. Previously, he merely complained of severe pain in the head.

case, disease of the alimentary canal seems to have induced hepatization of the lungs; and this latter to have led to the effusion of water into the brain, and considerable disorganization of this organ. Thus, we discover a primary, and perhaps trifling, indisposition of one part ultimately exerting a fatal, though gradual, influence, through a succession of intervening diseases.*

Another subject for consideration, and of no small importance, is whether a more active treatment than what appears to have been adopted might have proved more successful. I am strongly inclined to support this opinion.† There was, no doubt, a great tendency to inflammatory action, and, had measures calculated to reduce the phlogistic diathesis been instituted, and vigorously prosecuted, I must confess that I should anticipate a very different result. The means of counteracting the inflammatory tendency, are too well understood to require any comment in this place. However, there is one means for the attainment of this object, which seems to be much neglected by practitioners,—namely, *regimen;* under which term I comprehend clothing, diet, air, and exercise.

In such a case as the above, perhaps active depletion, frequently repeated, was not exactly indicated. A permanent drain, by a seton or issue, I should conceive better suited to the circumstances of the patient, and more adapted to control that languid inflammatory action, to which the system and the different organs seemed disposed. The inflammatory tendencies were not of that active description which require large and repeated venesection. The habit was to be broken rather by gradual, but permanent, operations. Regimen, in the comprehensive acceptation of the term, and issues, present the most favourable prospect in similar cases. I know a lady, who at an early period of life was attacked with severe hemoptysis, of a very serious nature, and considerable apprehensions were entertained for her safety. An issue was inserted in the arm, which checked the hemoptoe; and, about two years ago, I saw her in perfect health.‡ There was a scrofulous tendency in the

* The great advantage of a general examination of the body, must be very apparent from the above statement. The head was the part which I suspected to be diseased, and the brain was that first examined. The brain was found sufficiently diseased to account for the fatal event. By many, perhaps, the morbid inquiry would have been here terminated. But it must be evident, whether the views which I have advanced be admitted or not, that the examination of the brain alone would have given but a very limited and indistinct view of the nature and extent of the morbid changes in the different structures; and, consequently, the pathology of the case would have been but very imperfectly understood. I would, in all cases of post-mortem inquiry, examine all the vital organs, and the viscera of the abdomen.

† I could not learn that any active antiphlogistic measures had been adopted.

‡ I cannot say whether the issue still continues open.

family, and great fears were entertained lest the disease should terminate in phthisis pulmonalis.

Of course, the due regulation of the bowels, and attention to the digestion, would be objects of the utmost moment in the treatment of such cases. Whenever, from a scrofulous taint, the brain, the lungs, or any other part, is debilitated in its structure, the excitement and vascular activity arising from obstructed bowels or morbid digestion, will certainly induce diseased action in these parts, unless the morbid condition of the alimentary canal be removed, and the deranged state of the digestion be corrected.

Such are the reflections which, from a most careful and attentive review of the history and pathology of this case, have occurred to me. The facts I have candidly stated,* and faithfully recorded: the reader will determine for himself, whether the inferences be legitimately deducible.

*Henley-upon-Thames; July 31, 1824.*

* I was assisted in this dissection by Mr. Moran, (surgeon and assistant to Mr. Brooks, of this town,) Mr. Brooks, jun. and Mr. Young.

