# Errata in Our Last Number

**Published:** 1824-10

**Authors:** 


Errata *in our last Number.*

Page 195, line 9th of the note, for *periodide* read *protiodide.*
— 196, — 9 from the top, for *anticus* read *arterius.*

